# Thioflavin T as an efficient fluorescence sensor for selective recognition of RNA G-quadruplexes

**DOI:** 10.1038/srep24793

**Published:** 2016-04-21

**Authors:** Shujuan Xu, Qian Li, Junfeng Xiang, Qianfan Yang, Hongxia Sun, Aijiao Guan, Lixia Wang, Yan Liu, Lijia Yu, Yunhua Shi, Hongbo Chen, Yalin Tang

**Affiliations:** 1 National Laboratory for Molecular Sciences, Centre for Molecular Sciences, State Key Laboratory for Structural Chemistry of Unstable and Stable Species, Institute of Chemistry Chinese Academy of Sciences, Beijing, 100190, P. R. China; 2 University of the Chinese Academy of Sciences, Beijing, 100049, P. R. China

## Abstract

RNA G-quadruplexes (G4s) play important roles in translational regulation, mRNA processing events and gene expression. Therefore, a fluorescent probe that is capable of efficiently recognizing RNA G-quadruplex structures among other RNA forms is highly desirable. In this study, a water-soluble fluorogenic dye (i.e., Thioflavin T (ThT)) was employed to recognize RNA G-quadruplex structures using UV–Vis absorption spectra, fluorescence spectra and emission lifetime experiments. By stacking on the G-tetrad, the ThT probe exhibited highly specific recognition of RNA G-quadruplex structures with striking fluorescence enhancement compared with other RNA forms. The specific binding demonstrates that ThT is an efficient fluorescence sensor that can distinguish G4 and non-G4 RNA structures.

One of the most important types of nucleic acid structure is the G-quadruplex (G4), which is formed from four guanine bases by stacking of Hoogsteen bonded G-quartets^1^. The G-quadruplexes play a vital role in the human genome and transcriptome^2^,^3^. The DNA or RNA G-quadruplexes that are formed in cells are associated with many important cellular processes^4^,^5^,^6^,^7^,^8^. G-quadruplexes have been viewed as emerging therapeutic targets due to their correlation with human diseases^8^,^9^,^10^,^11^,^12^. Most of the early studies of G-quadruplexes focused on DNA strands, and few studies focused on RNA G-quadruplexes. Recently, RNA G-quadruplexes have been associated with many biological processes, such as telomere maintenance, pre-mRNA splicing and polyadenylation, RNA turnover, mRNA targeting and translation^13^. Therefore, the development of techniques that could efficiently recognize RNA G-quadruplexes structures and investigate their biological functions and impacts is highly desirable. Many techniques including X-ray crystallography and NMR experiments have been utilized to identify high-resolution structures of RNA G-quadruplexes^14^,^15^. However, these techniques are more suitable for comprehensively studying targeted RNA G-quadruplex structures. Additionally, circular dichroism (CD) has been extensively used to monitor G-quadruplex formation because the positive band at 264 nm and the negative band at 240 nm indicate the formation of parallel-type G-quadruplex structures^16^. However, it is difficult to interpret the G-quadruplex type in the presence of different forms of nucleic acids. Because the probe recognition sites in RNA G-quadruplexes are different from those in other RNA motifs, small molecules that selectively bind to RNA G-quadruplexes and emit fluorescence may be used as RNA G-quadruplex detectors. Our previous study revealed a cyanine dye (CyT) that was able to selectively recognize RNA G-quadruplex structures with ~2000-fold fluorescence enhancement^17^. Due to the important biological functions of RNA G-quadruplex, the development of a multiband probe that can selectively recognize RNA G-quadruplex structures is needed to further expand the application of RNA G-quadruplexes, which have been recognized as significant molecular targets. Then, Thioflavin T (ThT, Fig. 1), which is an extrinsic fluorescent probe for the identification of amyloid fibrils in previous studies^18^,^19^, was selected to target RNA G-quadruplex using a high-throughput screening approach. Recently, ThT was reported to recognize the human telomeric motif and was used to discriminate this structure from other DNA forms^20^,^21^. In this study, we employed ThT as a fluorescent probe to target RNA G-quadruplexes (Table 1). The RNA G-quadruplex structure-selective binding of ThT was employed to recognize RNA G-quadruplex structures from other RNA forms via its fluorescence light-up (Fig. 2). The fluorescence intensity enhancement, which results from ThT specifically binding to RNA G-quadruplex, is expected to provide another RNA G-quadruplex probe.

## Results

### ThT selectively targeted RNA G-quadruplexes with fluorescence enhancement and red shift

**Figure 3:**
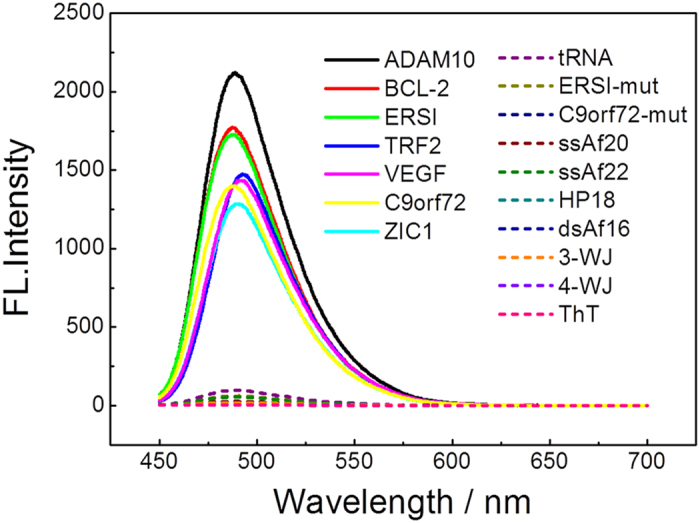
Fluorescence emission spectra of 2 μM ThT with various oligonucleotides (4 μM) in a 20 mM Tris HCl (40 mM K^+^, pH 7.0) solution.

**Figure 4:**
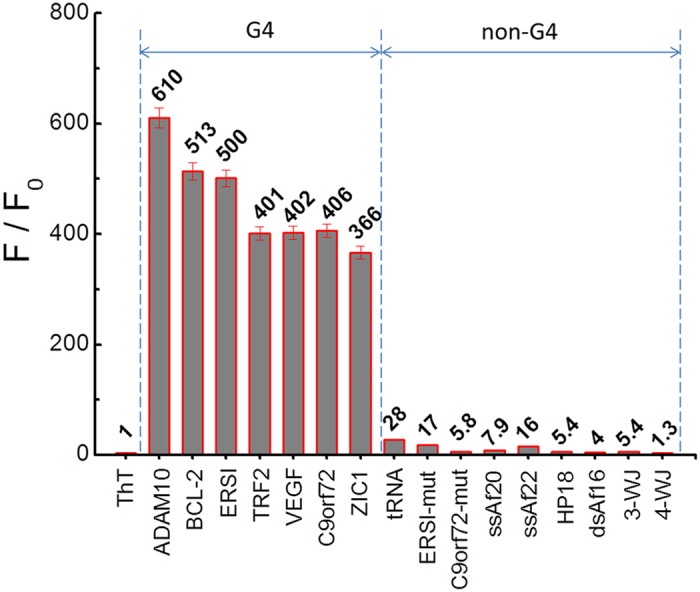
Dependence of ThT (2 μM) fluorescence intensity at 487 nm for a variety of RNA sequences (4 μM) in a 20 mM Tris HCl (40 mM K^+^, pH 7.0) solution.

To investigate the feasibility of the ThT probe for RNA G-quadruplex structure recognition, we examined its selectivity towards various RNA forms including RNA G-quadruplex sequences (ADAM10, BCL-2, ERSI, TRF2, VEGF, C9orf72 and ZIC1) and other RNA forms, such as transfer RNA (tRNA), mutation sequences (ERSI-mut, C9orf72-mut), single-stranded (ssAf20, ssAf22), hairpin (HP18), double-stranded (dsAf16), three-way junction (3-WJ) and four-way junction (4-WJ). As shown in Fig. 3, the ThT probe exhibited a very weak emission at 487 nm when diluted in pH 7.0 Tris HCl buffer. In addition, ThT is weakly emissive in the presence of other RNA forms. Surprisingly, remarkable fluorescence enhancements were obtained after the addition of RNA G-quadruplex sequences, and the F/F_0_ was determined to be as large as 610-, 513-, 500-, 401-, 402-, 406- and 366-fold for ADAM10, BCL-2, ERSI, TRF2, VEGF, C9orf72 and ZIC1, respectively (Fig. 4). The observed fluorescence enhancements were due to the specific binding of the ThT probe with RNA G-quadruplex structures. Our results suggest that ThT can be used as a fluorophore to target RNA G-quadruplex structures with an accompanying fluorescence enhancement.

Because ThT selectively binds to G-quadruplex DNA in the human telomeric motif, we further compared the recognition of the ThT probe for the detection of RNA G-quadruplexes and DNA G-quadruplexes. As shown in Supplementary Fig. S1, ThT has a selective fluorescence response for RNA G-quadruplex structures compared to the response to other RNA forms. The fluorescence enhancements of the RNA G-quadruplex sequences (Tel22, BCL-2 and C9orf72) were 440-, 513- and 406-fold, respectively, which were substantially higher than those with other RNA forms (ssAf20, HP18 and dsAf16). The fluorescence enhancements of ssAf20, HP18 and dsAf16 at 487 nm were only 7.9-, 5.4- and 4-fold, respectively. When the ThT probe interacted with different DNA forms (see Supplementary Fig. S2), the fluorescence enhancements of human telomeric DNA G-quadruplex (Tel22) and the DNA G-quadruplex in the promoter region (BCL-2, C9orf72) were 1339-, 458- and 252-fold, respectively. However, the results indicated that ThT can also interact with non-G4 DNA forms, such as single-stranded ssAf20 (59-fold), hairpin motifs HP18 (36-fold) and double-stranded dsAf16 (65-fold), which demonstrates poor selectivity for DNA G-quadruplex structures with respect to other DNA forms.

**Figure 5:**
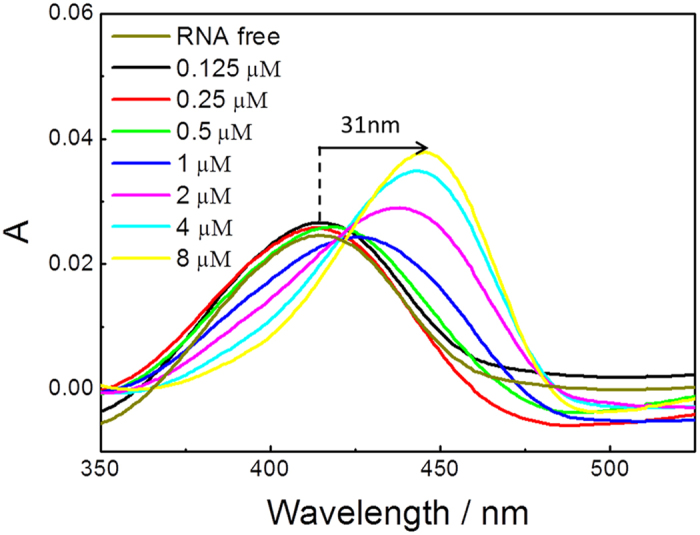
Absorption spectra of ThT (2 μM) with RNA G-quadruplex sequence (ADAM10) at eight concentrations (μM): (i) 0, (ii) 0.125, (iii) 0.25, (iv) 0.5, (v) 1, (vi) 2, (vii) 4 and (viii) 8.

Furthermore, the specific binding of ThT with RNA G-quadruplex structures was further demonstrated by the absorption spectra. As shown in Fig. 5, gradual addition of the RNA G-quadruplex sequence (with ADAM10 sequence as an example, from 0.125 μM to 8 μM) to the ThT solution induced a red shift from 414 nm to 445 nm, which indicated the specific interaction of the ThT probe with the RNA G-quadruplex structure. The red shift in the ThT absorption may arise from ligand binding to the RNA G-quadruplex structure, which prevents rotation of the benzothiazole ring relative to the aminobenzene ring in the excited state^22^.

**Figure 6:**
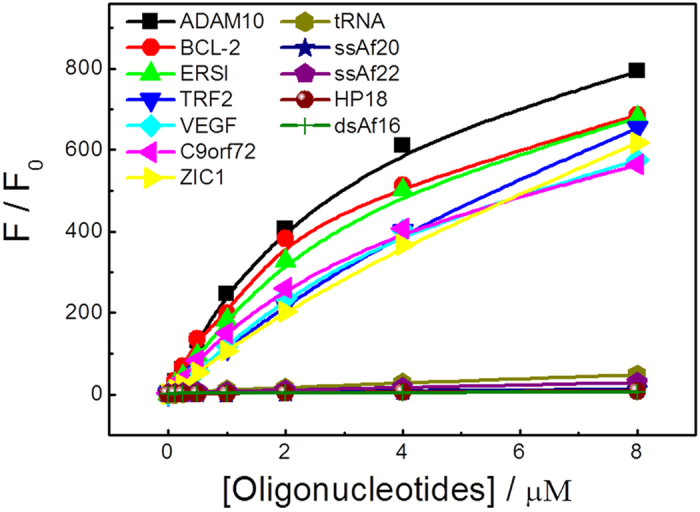
Fluorescence intensity enhancement (F/F_0_) of ThT (2 μM) at 487 nm plotted as a function of various RNA forms at different concentrations (from 0.125 μM to 8 μM). The solid lines are the fitted curves assuming 1:1 stoichiometry.

Fluorescence titrations of ThT with various RNA forms were conducted to evaluate the binding constants. The typical titration curves and fitting results are presented in Fig. 6. A 1:1 stoichiometric analysis (confirmed by a Job’s plot analysis in this study, as shown in Supplementary Fig. S3) of this binding curve provided the binding constants for ThT with ADAM10, BCL-2, ERSI, TRF2, VEGF, C9orf72 and ZIC1, which are (2.6 ± 0.12) × 10^5^, (2.93 ± 0.27) × 10^5^, (2.03 ± 0.12) × 10^5^, (0.62 ± 0.61) × 10^5^, (1.24 ± 0.15) × 10^5^ M^−1^, (1.92 ± 0.05) × 10^5^ M^−1^ and (0.56 ± 0.22) × 10^5^ M^−1^, respectively. Additionally, binding constants of (5.51 ± 0.83) × 10^4^, (9.37 ± 1.28) × 10^4^ M^−1^ (5.53 ± 1.59) × 10^4^, (5.62 ± 1.94) × 10^4^, and (1.43 ± 1.01) × 10^5^ for ThT with tRNA, ssAf20, ssAf22, HP18 and dsAf16, respectively, were also obtained (see Supplementary Table S1). In comparison to RNA G-quadruplex structures, the binding strength to these sequences was much weaker. It is important to note that although ThT does exhibit a reasonable binding affinity with dsAf16, the dye may have flexible orientations at the site and not exhibit fluorescence enhancement^23^.

### Monitoring the RNA G-quadruplex structures by fluorescence lifetime measurements

**Figure 7:**
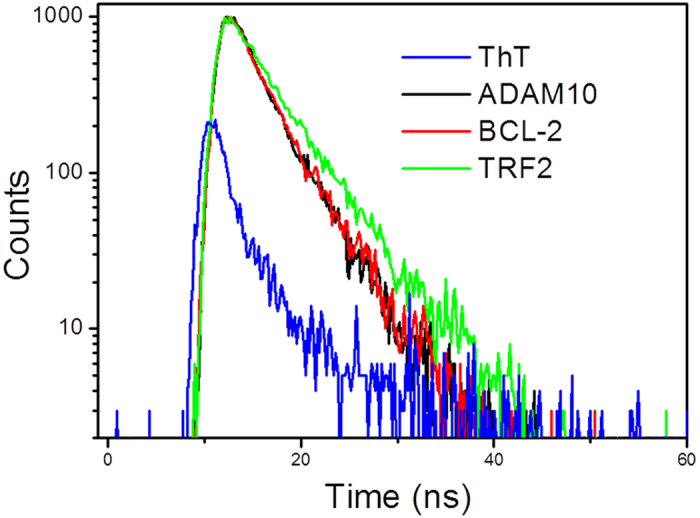
Fluorescence decay traces of ThT (2 μM) in the absence and presence of RNA G-quadruplex structures (4 μM). The samples were prepared in a 20 mM Tris HCl (40 mM KCl, pH 7.0) buffer solution.

Based on the emission enhancement and the absorption spectral results, fluorescence intensity decay measurements were conducted to demonstrate the strong binding of ThT with the RNA G-quadruplex structures. The fluorescence decay of ThT was very fast (<1 ps)^24^ in solution due to the highly feasible torsional relation channel in the exited state. However, the lifetimes significantly increased to the nanosecond level in the presence of RNA G-quadruplex structures. As shown in Fig. 7, the fluorescence decay was much lower for ThT in the presence of ADAM10, BCL-2 and TRF2 (i.e., 3.69 ns, 3.66 ns and 4.68 ns, respectively). This result dramatically changes of the lifetime of ThT due to the contribution from the strong interaction between ThT and RNA G-quadruplex structures, which hinders the rotation of ThT and increases the lifetime.

### ThT is likely to interact with RNA G-quadruplex via the end-stacking mode

To explore the mechanisms of ThT binding with the RNA G-quadruplex structure, molecular modelling studies of the 1:1 complex of RNA G-quadruplex with ThT have been performed. In general, the RNA G-quadruplex structures have several sites of interaction with the probes (i.e., end-stacking and groove binding modes). The molecular docking results indicate that the binding free energy of the end-stacking mode (−8.56 kcal mol^−1^) was much lower than that of the groove binding mode (−6.32 kcal mol^−1^). As shown in Supplementary Fig. S4a, the ThT probe interacts with the RNA G-quadruplex by stacking with the top G-tetrad of the RNA G-quadruplex structure. The benzothiazole moieties in ThT stacked onto the top G-tetrad and contributed most of the pi-stacking force in probe binding. The methyl group at the N3 position on the benzothiazole ring of ThT is located at the centre of the top G-tetrad and matches the size of the top G-tetrad central channel of the RNA G-quadruplex structure. Based on these results, ThT is more likely to interact with RNA G-quadruplex by the end-stacking mode on the top G-tetrad.

### ThT as an efficient probe for the recognition of non-canonical RNA G-quadruplex structures

Recent structural and biophysical studies have provided some methods for identifying G-quadruplex secondary structures. Canonical G-quadruplex secondary structures are formed by four tracts of three or more continuous guanines through hydrogen bonding. However, recent studies have demonstrated that nucleic acids do not obey the classical description of G_2+_N_L1_G_2+_N_L2_G_2+_N_L3_G_2+_ sequences may also form G-quadruplex structures^25^,^26^,^27^,^28^, in which either the discontinuous arrangement of guanines in the G-tetrad core leads to Spinach^26^ and singe-nucleotide interruptions in one or more of the G-runs or a longer interruption in one G-run leads to Bulges-TB1^25^. In addition, the tRNA-Cys fragment and tRNA-Ala fragment^29^ (but not full length tRNA) assemble G-quadruplex structures. However, these non-canonical G-quadruplex structures could not have been easily identified by the classical approaches for G-quadruplex formation. Therefore, to further extend the application of RNA G-quadruplex structures in biological function and cancer development, an effective and universal detector is expected to detect more RNA G-quadruplex forming sequences that have not been identified by the previous methods. The ThT probe may be able to recognize the non-canonical RNA G-quadruplex structures. As a result, non-canonical RNA G-quadruplexes including Spinach, Bulges-TB1 and tRNA-fragment (tRNA-Cys-fragment and tRNA-Ala-fragment) have been used to bind to the ThT probe.

**Figure 8:**
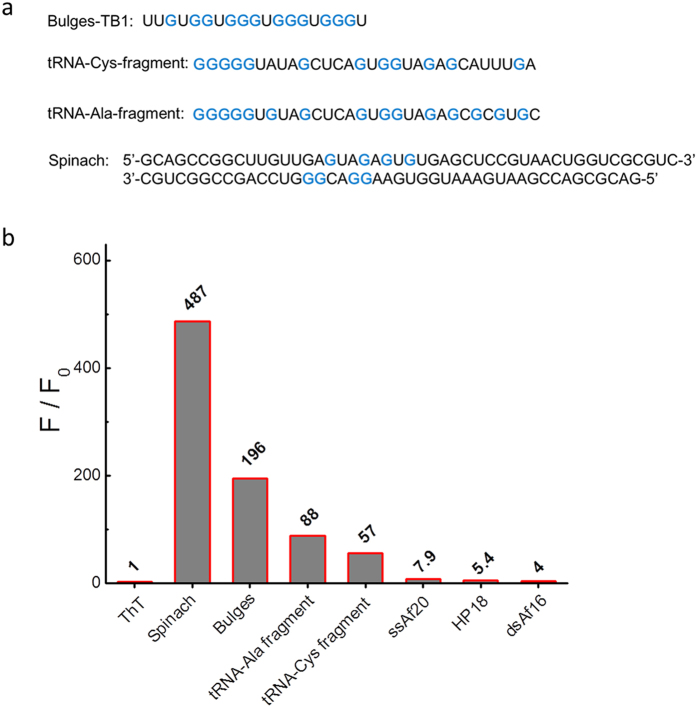
(**a**) Sequences of non-canonical G-quadruplexes used in this study. G-bases that are marked in blue were involved in forming the RNA G-quadruplex structure. (**b**) Dependence of the ThT (2 μM) fluorescence intensity in 20 mM Tris HCl buffer (40 mM K^+^, pH 7.0) on the non-canonical G4 sequences and non-G4 sequences (4 μM).

Four non-canonical RNA G-quadruplexes, Spinach, Bulges-TB1, tRNA-Ala-fragment and tRNA-Cys-fragment (Fig. 8a) were employed to bind to the ThT probe, and single-stranded RNA (the ssAf20), hairpin motifs RNA (the HP18) and double-stranded RNA (the dsAf16) were tested as non-G4 RNA controls. As shown in Fig. 8b, the F/F_0_ is determined to be approximately 487-, 196-, 88- and 57-fold for Spinach, Bulges-TB1, tRNA-Ala-fragment and tRNA-Cys-fragment, respectively, which are much higher than those for the non-G4 RNA controls. It important to note that the fluorescence enhancement was lower for some of the non-canonical RNAs than for the canonical ones due to the lower stability of non-canonical RNA G-quadruplex structures, which exhibit discontinuous G-tracts in the G-tetrad core formation. However, the ThT can still precisely distinguish non-canonical RNA G-quadruplexes from other RNA forms. This approach is applicable to the study of G-quadruplex prevalence in any given genome. Then, the ThT probe may also be extended to the detection of more RNA G-quadruplex structures in many parts of the genome, providing a resource of genomic targets for further mechanistic studies and therapeutic intervention.

### DNA G-quadruplex probe (Thiazole Orange as an example) may not be suitable for RNA G-quadruplex detection without validation

**Figure 9:**
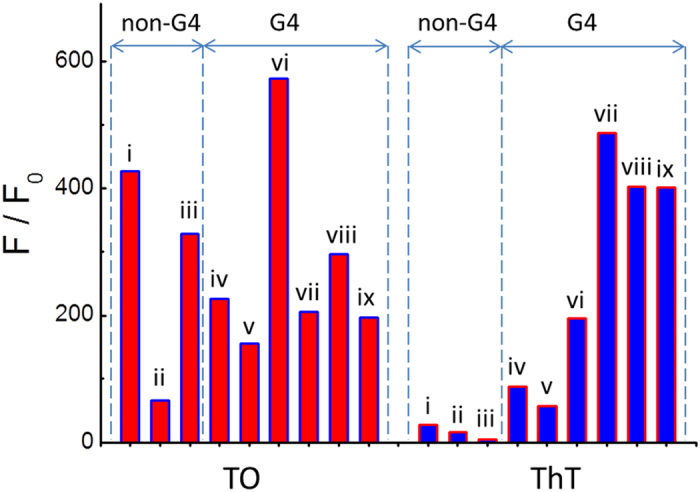
Dependence of the TO and ThT fluorescence intensity on the G4 and non-G4 sequences in a 20 mM Tris HCl (40 mM K^+^, pH 7.0) solution. (i) tRNA, (ii) ssAf22, (iii) HP18, (iv) tRNA-Ala fragment, (v) tRNA-Cys fragment, (vi) Bulges-TB1 (vii) Spinach, (viii) VEGF and (ix) TRF2.

Due to the structural similarities of DNA and RNA G-quadruplex, Thiazole Orange (TO), which is a well-known DNA G-quadruplex binding dye^30^, was employed to explore the selectivity to RNA G-quadruplex structures. To determine the effect on the RNA G-quadruplex structure of DNA G-quadruplex ligands, the fluorescence measurements of TO were carried out in the presence of different RNA forms including canonical RNA G-quadruplex sequences (VEGF, TRF2), non-canonical RNA G-quadruplex sequences (RNA-Ala fragment, tRNA-Cys fragment, Bulges-TB1, Spinach), control sequences (ssAf22, HP18) and tRNA. The results are shown in Fig. 9, and TO exhibited a fluorescence enhancement of approximately 427-, 65-, 328-, 226-, 156-, 200-, 573-, 206-, 295- and 197-fold for (i) tRNA, (ii) ssAf22, (iii) HP18, (iv) tRNA-Ala fragment, (v) tRNA-Cys fragment, (vi) Bulges-TB1 (vii) Spinach, (viii) VEGF and (ix) TRF2, respectively. The DNA G-quadruplex ligand does not exhibit any selectivity for RNA G-quadruplex. Therefore, an efficient probe for DNA G-quadruplex may not be suitable for recognizing RNA G-quadruplex structures without validation.

## Discussion

According to these results, we discovered that ThT, which is a water-soluble fluorogenic dye with low self-fluorescence, exhibited substantially fluorescence enhancement in the presence of RNA G-quadruplexes compared to the fluorescence enhancement of less than 30-fold that was observed in the presence of non-G4 RNA. The substantial increase in the fluorescence emission was due to restricting the rotation and enforcing the planarization of the dye^22^,^31^. Its specific binding with RNA G-quadruplex structures allows for the development of an efficient method for the detection of RNA G-quadruplexes and provides a promising tool for RNA G4-based biomarker discovery with potential diagnostic applications. Additionally, the ThT probe may be used to identify non-canonical RNA G-quadruplex formation. In general, in G-quadruplex formation, continuous G-tracts are needed in the sequence. Although recent studies have demonstrated that nucleic acids obeying the classical description with G_2+_N_L1_G_2+_N_L2_G_2+_N_L3_G_2+_ sequences may also form G-quadruplex structures^25^,^26^,^27^,^28^. In previous studies, potential canonical RNA G-quadruplex forming sequences have been detected using bioinformatics searches. However, some non-canonical potential RNA G-quadruplex forming sequences have not been detected using this method. Therefore, an effective and universal detector is needed to identify non-canonical RNA G-quadruplex structures. In comparison to canonical RNA G-quadruplex, the lower fluorescence obtained with non-canonical RNA G-quadruplex structures may be due to the lower stability of non-canonical RNA G-quadruplex, which has as many as three isolated guanines.

Furthermore, TO and ThT are widely used to selectively target DNA G-quadruplex. However, our results indicate that thiazole orange (TO) does not exhibit any selectivity for RNA G-quadruplexes compared to other RNA forms. Therefore, a DNA G-quadruplex probe may not be suitable for detecting or recognizing RNA G-quadruplexes without validation. ThT has been demonstrated to act as a universal probe to recognize RNA G-quadruplex or DNA G-quadruplex structures. However, the G-quadruplex structure formed from human telomeric DNA is different from the G-quadruplex structure formed from RNA. The G-quadruplex in a human telomeric motif is typically a stabilized hybrid structure, and the RNA G-quadruplexes formed a parallel-stranded conformation regardless of the different sequences and the experimental conditions^32^. Additionally, the ThT probe exhibited better selectivity for RNA G-quadruplex structures than DNA G-quadruplex structures. The ability of ThT to distinguish between G4 and non-G4 RNA/DNA offers a promising tool for future G4-based biomarker discovery with the potential for diagnostic applications. This study allows for the exploration of fluorogenic dyes based on ThT, which have the potential to emerge as highly specific quadruplex sensing agents for diagnostic and therapeutic applications.

## Materials and Methods

### Oligonucleotides and compounds

The oligonucleotide sequences that are shown in Table 1 were synthesized by RiboBio Co., Ltd (Guangzhou, Guangdong, China). Transfer RNA (Ribonucleotide acid; transfer from bovine liver; R4752) was purchased from Sigma-Aldrich. The RNA stock solutions were prepared by dissolving oligonucleotides directly in 20 mM Tris HCl at a pH of 7.0 with 40 mM KCl followed by annealing in a thermocycler (first heated at 90 °C for 2 min and then cooled down slowly to room temperature).

3,6-Dimethyl-2-(4-dimethylaminophenyl)-benzothiazolium cation (ThT) and the thiazole orange (TO) dyes were purchased from Sigma-Aldrich and used without further purification. The stock solutions of ThT and TO were prepared by dissolving in water. All of the other chemicals were of analytical reagent grade and used without further purification. Ultrapure water, which was prepared using a Milli-Q Gradient ultrapure water system (Millipore), was used in all of the experiments.

### Absorption spectroscopy

The absorption spectra were acquired with a UV-1601PC at room temperature using a quartz cell with a path length of 10 mm. The absorption titration experiments were performed by increasing the RNA G-quadruplex concentrations from 0.125 to 8 μM, and ThT was fixed at 2 μM.

### Fluorescence single wavelength measurements

The experiments were carried out with 96-well microplates from CORNING (Flat Bottom Black Polystyrol). The RNA samples and ThT were mixed at 4 μM and 2 μM final concentrations, respectively, in 20 mM Tris HCl at a pH of 7.0 and 40 mM KCl. The measurements were performed at room temperature. The fluorescence emission was collected at 487 nm with excitation at 440 nm in a microplate reader (PE EnSpire).

### Fluorescence spectroscopy

The fluorescence spectra were acquired with a Hitachi F-4500 spectrophotometer at 25±1 °C, which was equipped with a temperature-controlled circulator. A quartz cuvette with a 10-mm path length was used in all of the experiments. In the fluorescence measurements, both the excitation and emission slits were 5 nm, and the scan speed was 240 nm/min. ThT was titrated with RNA G-quadruplex to measure the binding constants, and the fluorescence intensity at 440 nm was plotted as a function of the RNA concentration. The data were fitted according to a 1:1 binding model. The titration experiments were performed by increasing the RNA G-quadruplex concentrations from 0.125 to 8 μM ThT, which was added to the different RNA concentration solutions with gentle stirring for 10 min, and then, the samples were maintained in darkness for 2 hours prior to the measurements.

The fluorescence lifetime measurements were recorded on a time-correlated single photon counting FLSP920 system. From the measured decay traces, the data were fitted with a multi-exponential decay, and χ^2^ was less than 1.1.

### Molecular Modelling

The coordinates of the RNA quadruplex (3IBK) structures were retrieved from the RSCB Protein Data Bank. The RNA quadruplex structure was prepared for molecular docking, and the ThT structure was energy minimized (MMFF force field) using the Discovery Studio 3.5 program (Accelrys Software Inc., San Diego). The molecular docking studies were performed using the Lamarckian Genetic Algorithm as implemented in the Autodock 4.0 package. The binding site (docking grids) was prepared using 100 × 100 × 100 points with a grid spacing of 0.375 Å. For the Lamarckian Genetic Algorithm based docking, the size of the population was set to 150, and the number of energy evaluations was set to 5.0 × 10^7^. All of the figures were rendered using the Discovery Studio 3.5 program (Accelrys Software Inc., San Diego).

## Additional Information

**How to cite this article**: Xu, S. *et al*. Thioflavin T as an efficient fluorescence sensor for selective recognition of RNA G-quadruplexes. *Sci. Rep*. **6**, 24793; doi: 10.1038/srep24793 (2016).

## Supplementary Material

Supplementary Information

## Figures and Tables

**Figure 1:**
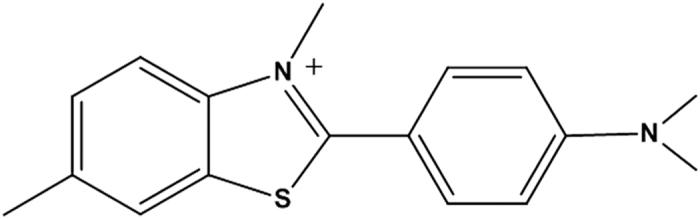
Molecular structure of ThT.

**Figure 2:**
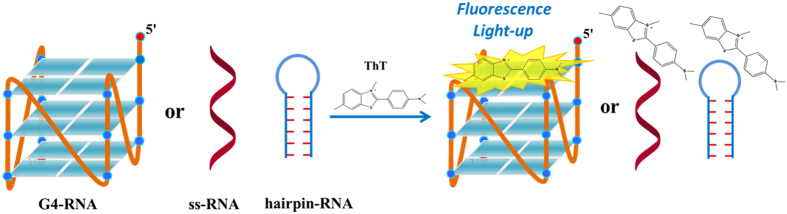
Schematic representations of ThT binding with various RNA forms for G-quadruplex structure recognition. The fluorescence intensity was substantially enhanced by addition of RNA G-quadruplex sequences, and the ss-RNA and hairpin RNA do not induce enhanced fluorescence.

**Table 1:** Oligonucleotides used in the study.

Name	Type/origin	Sequence (from 5′ to 3′)	Ref.
ADAM10	Canonical G4-5**′**-UTR	GGGGGACGGGUAGGGGCGGGAGGUAGGGG	33
BCL-2	Canonical G4-5**′**-UTR	AGGGGGCCGUGGGGUGGGAGCUGGGG	34
ERSI	Canonical G4-5**′**-UTR	GGGUAGGGGCAAAGGGGCUGGGG	35
TRF2	Canonical G4-5**′**-UTR	CGGGAGGGCGGGGAGGGC	36
ZIC1	Canonical G4-5**′**-UTR	GGGUGGGGGGGGCGGGGGAGGCCGGGG	37
VEGF	Canonical G4-5**′**-UTR	GGAGGAGGGGGAGGAGGA	38
C9orf72	Canonical G4	GGGGCCGGGGCCGGGGCCGGGGCC	39
Tel22	Canonical G4-TERRA	AGGGUUAGGGUUAGGGUUAGGG	
BCL-2	Canonical DNA G4	AGGGGGCCGTGGGGTGGGAGCTGGGG	
C9orf72	Canonical DNA G4	GGGGCCGGGGCCGGGGCCGGGGCC	
Tel22	Canonical DNA G4	AGGGTTAGGGTTAGGGTTAGGG	
Bulges-TB1	Non-canonical RNA G4	UUGUGGUGGGUGGGUGGGU	25
Spinach	Non-canonical RNA G4	GCAGCCGGCUUGUUGAGUAGAGUGUGAGCUCCGUAACUGGUCGCGUC	26
		GACGCGACCGAAUGAAAUGGUGAAGGACGGGUCCAGCCGGCUGC	
tRNA-Ala fragment	tRNA fragments	GGGGGUGUAGCUCAGUGGUAGAGCGCGUGC	29
tRNA-Cys fragment	tRNA fragments	GGGGGUAUAGCUCAGUGGUAGAGCAUUUGA	29
ERSI-mut	Mutation	GUGUAGUUGCAAAGUGUCUGUGG	–
C9orf72-mut	Mutation	GUGGCCGUGGCCGUGGCCGUGGCC	–
tRNA	Transfer RNA		–
ssAf20	Single strand RNA	CAAUUGUAUAUAUUCG	–
ssAf22	Single strand RNA	UGAGCUUAAUUGUAUAUAUUCG	–
HP18	Hairpin RNA	CAGUACAGAUCUGUACUG	4
dsAf16	Duplex RNA	CUUAAUUGUAUAUAUUCGCGAAUAUAUACAAUUAAG	–
ssAf20	Single strand DNA	CAATTGTATATATTCG	
HP18	Hairpin DNA	CAGTACAGATCTGTACTG	
dsAf16	Duplex DNA	CTTAATTGTATATATTCGCGAATATATACAATTAAG	
3-WJ	Three-way junction (entry ID 619)	AGCGCAACCCCUCGUCAGCUGGGACGACGU	40
4-WJ	Four-way junction (entry ID 1173)	GCGCUUUAGCGAGGUCCUAGAA	40
