# Supplementary material for: Thioflavin T as an efficient fluorescence sensor for selective recognition of RNA G-quadruplexes
Source: Sci Rep. 2016 Apr 21;6:24793. doi: 10.1038/srep24793 (PMC4838840; doi:10.1038/srep24793)
Supplement: Supplementary Information [file srep24793-s1.doc]

Supplementary Information

**Thioflavin T as an** **efficient fluorescence sensor for selective recognition of RNA G-quadruplex**

Shujuan Xu^1,2^, Qian Li^1,*^, Junfeng Xiang^1^, Qianfan Yang^1^, Hongxia Sun^1^, Aijiao Guan^1^, Lixia Wang^1^, Yan Liu^1^, Lijia Yu^1,2^, Yunhua Shi^1,2^, Hongbo Chen^1^, and Yalin Tang^1,*^

^1^ National Laboratory for Molecular Sciences, Center for Molecular Sciences, State Key Laboratory for Structural Chemistry of Unstable and Stable Species, Institute of Chemistry Chinese Academy of Sciences, Beijing, 100190, P. R. China

^2^ University of the Chinese Academy of Sciences, Beijing, 100049, P. R. China

* To whom correspondence should be addressed. Tel: + 86 10 62522090; Fax: +86 10 62522090; Email: tangyl@iccas.ac.cn. Correspondence may also be addressed to Qian Li. [Tel: + 86 10 62558322; Fax: +86 10 62558322; Email: qianlee@iccas.ac.cn](mailto:Tel: + 86 10 62558322; Fax: +86 10 62558322; Email: qianlee@iccas.ac.cn).


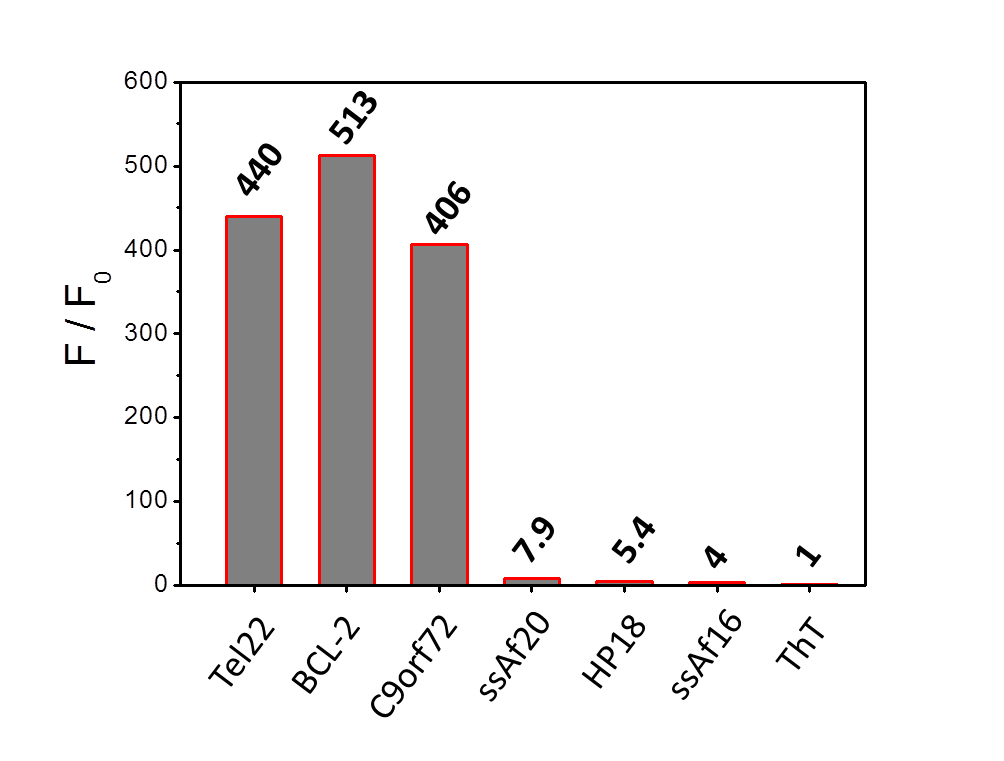


**Figure S1.** Dependence of ThT (2 µM) fluorescence intensity at 487 nm on a variety of RNA sequences (4 µM) in 20 mM Tris-HCl (40 mM KCl, pH 7.0) solution.


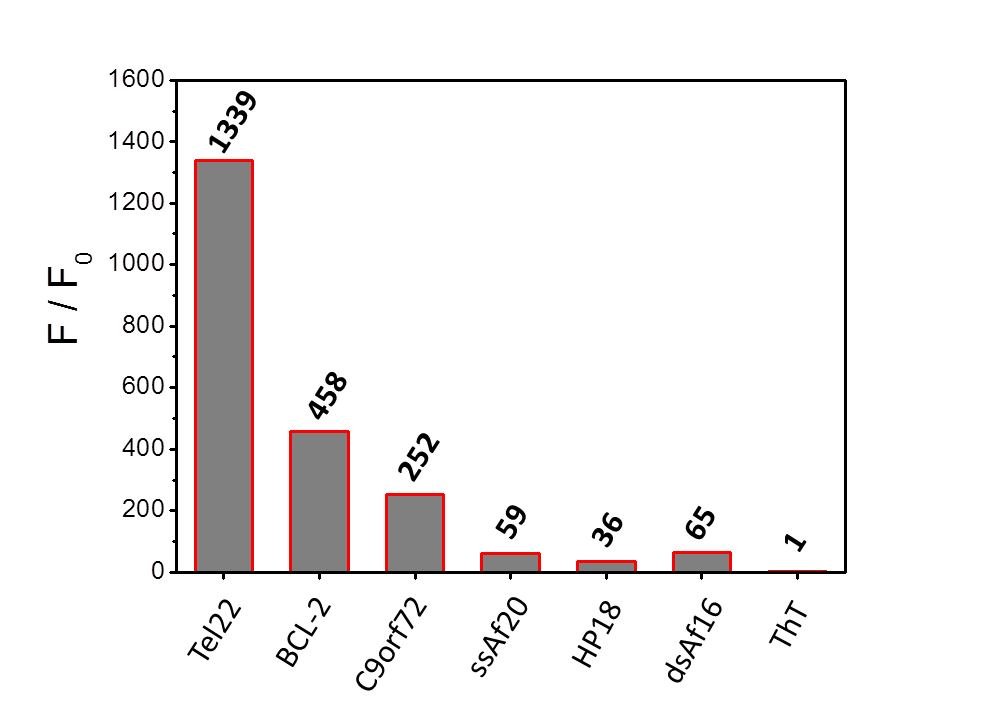


**Figure S2.** Dependence of ThT (2 µM) fluorescence intensity at 487 nm on a variety of DNA sequences (4 µM) in 20 mM Tris-HCl (40 mM KCl, pH 7.0) solution.

**
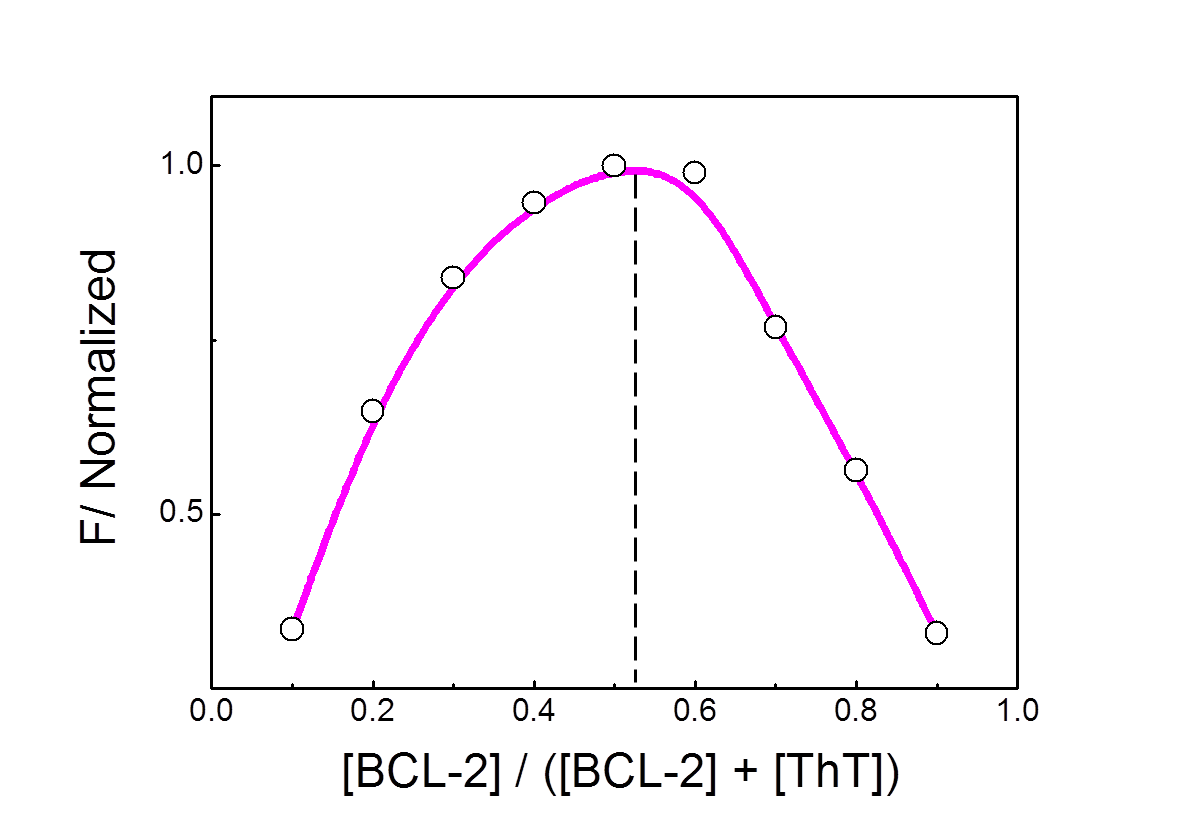
**

**Figure S3.** Job’s plot analysis of the stoichiometry of BCL-2 binding to ThT.


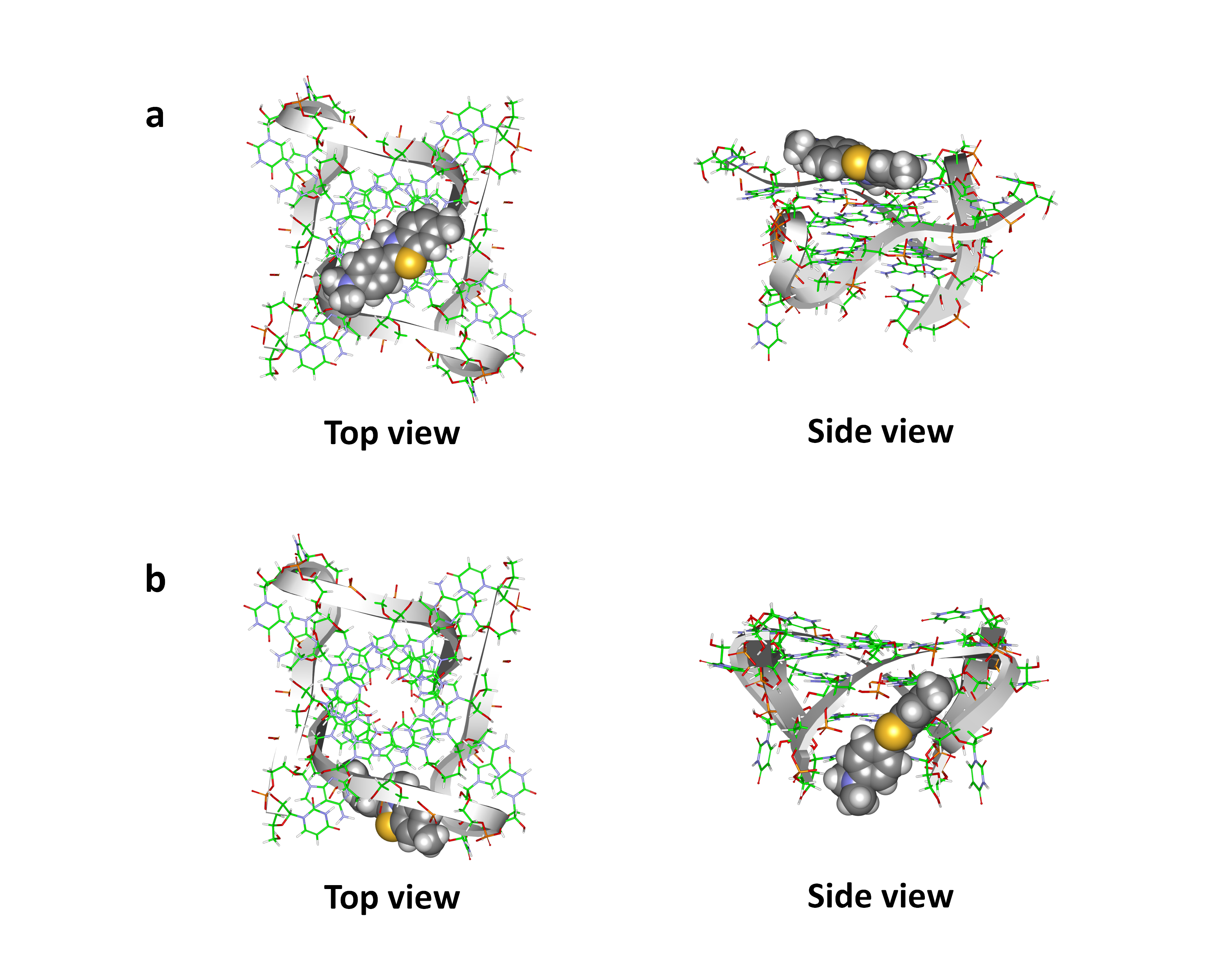


**Figure S4.** Molecular modeling results showing the interaction of ThT with RNA G-quadruplex structures by end-stacking mode (a) and groove-binding mode (b) The RNA G-quadruplex structure was shown by wire model and the ThT was shown by space-filling model.

**Table S1.** Binding constants for the ThT probe with different RNA forms determined from the fitted curves.

| RNA sequences | K (M^-1^) |
| --- | --- |
| ADAM10 | (2.60±0.12)×10^5^ |
| BCL-2 | (2.93±0.27)×10^5^ |
| ERSI | (2.03±0.12)×10^5^ |
| TRF2 | (0.62±0.61)×10^5^ |
| VEGF | (1.24±0.15)×10^5^ |
| C9orf72 | (1.92±0.05)×10^5^ |
| ZIC1 | (0.56±0.22)×10^5^ |
| tRNA | (5.51±0.83)×10^4^ |
| ssAf20 | (9.37±1.28)×10^4^ |
| ssAf22 | (5.53±1.59)×10^4^ |
| HP18 | (5.62±1.94)×10^4^ |
| dsAf16 | (1.43±1.01)×10^5^ |
